# Intestinal Malrotation: A Rare Cause of Small Intestinal Obstruction

**DOI:** 10.1155/2014/453128

**Published:** 2014-10-09

**Authors:** Mesut Sipahi, Kasim Caglayan, Ergin Arslan, Mustafa Fatih Erkoc, Faruk Onder Aytekin

**Affiliations:** ^1^Department of General Surgery, School of Medicine, Bozok University, 66100 Yozgat, Turkey; ^2^Department of Radiology, School of Medicine, Bozok University, 66100 Yozgat, Turkey

## Abstract

*Background*. The diagnosis of intestinal malrotation is established by the age of 1 year in most cases, and the condition is seldom seen in adults. In this paper, a patient with small intestinal malrotation-type intraperitoneal hernia who underwent surgery at an older age because of intestinal obstruction is presented. *Case*. A 73-year-old patient who presented with acute intestinal obstruction underwent surgery as treatment. Distended jejunum and ileum loops surrounded by a peritoneal sac and located between the stomach and transverse colon were determined. The terminal ileum had entered into the transverse mesocolon from the right lower part, resulting in kinking and subsequent segmentary obstruction. The obstruction was relieved, and the small intestines were placed into their normal position in the abdominal cavity. *Conclusion*. Small intestinal malrotations are rare causes of intestinal obstructions in adults. The appropriate treatment in these patients is placement of the intestines in their normal positions.

## 1. Introduction

Intestinal malrotation is a congenital disorder caused by rotation of the intestines during foetal development. Embryological development and anatomical variations were described in 1923 by Dott [1]. The intestines start to grow in the fourth week of gestation. Physiological herniation occurs in the umbilical cord causing it to rotate in an anticlockwise direction. The hernia is reduced in the 10th week of gestation, and the caecum settles in its normal right bottom position at the 12th week [2]. Intestinal malrotation is a disorder resulting from the lack of foetal intestinal physiological rotation [3]. There is often a fibrous band called Ladd's band that prevent the rotation of the intestines. Intestinal malrotations comprise various anatomic anomalies ranging from complete nonrotation to normal positioning [4, 5]. Intestinal malrotations are named according to anatomical variations such as incomplete rotation, mixed rotation, atypical malrotation, and variants of malrotation [6]. They can be categorised into two groups: typical and atypical malrotation based on the position of the ligament of Treitz according to the right and left of the midline, respectively [4]. Intestinal malrotations occur in approximately 0.2% of all births. Symptoms usually occur in the early weeks of life, and the malrotations are generally diagnosed during this period. More than 40% of intestinal malrotations are diagnosed within 1 week after birth and 75–85% within 1 year after birth [6]. Although the precise incidence of intestinal malrotation is unknown, it is estimated that it occurs between the rates of 0.0001% and 0.19% in adults [3]. Generally, intestinal malrotation is incidentally determined in adults due to its asymptomatic or nonspecific presentation with mild symptoms. In the present paper, we present a case of an elderly patient with intestinal malrotation-type intraperitoneal hernia. The colon was rotated normally, but all of the intraperitoneal small intestines were placed in the lesser sac.

## 2. Case Report

**Figure 1:**
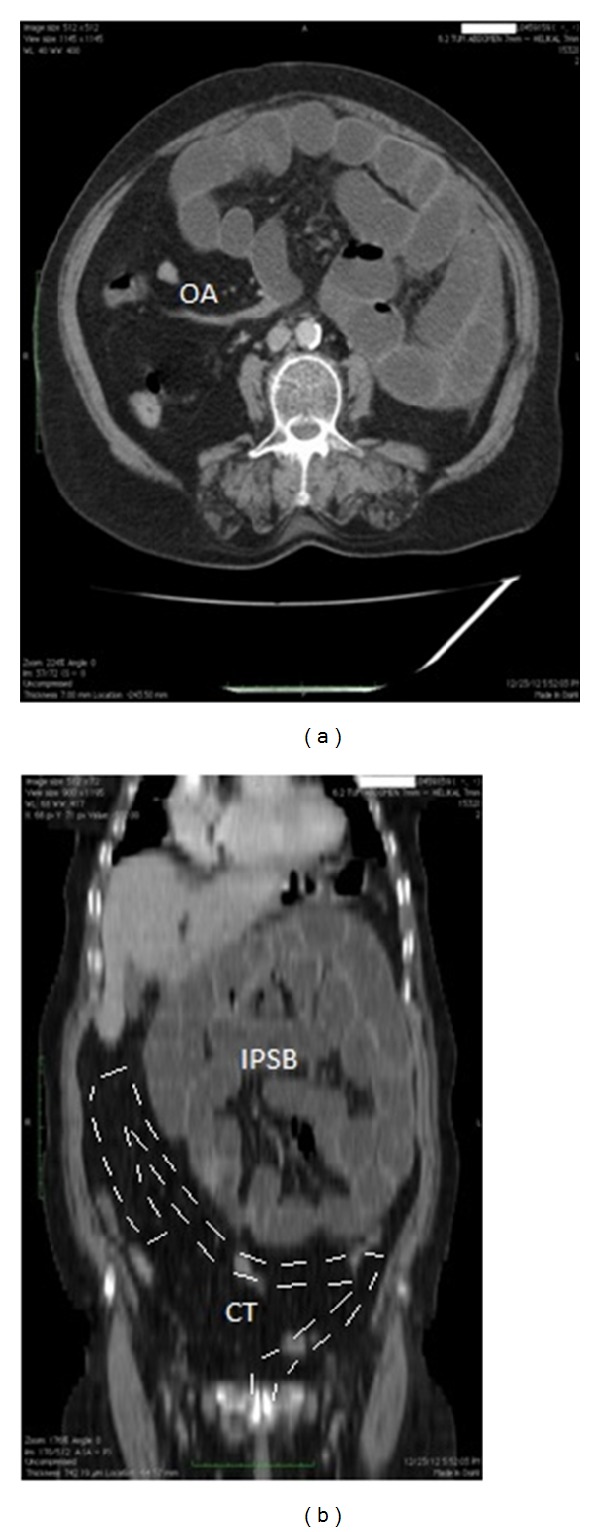
CT images demonstrating intestinal malrotation. On axial image (a), obstructed area (OA) indicates the obstructed part of terminal ileum. On coronal (b) image intraperitoneal small bowel (IPSB) is indicated in upper abdomen. Colon trace (CT) is oriented with (-) symbol.

**Figure 2:**
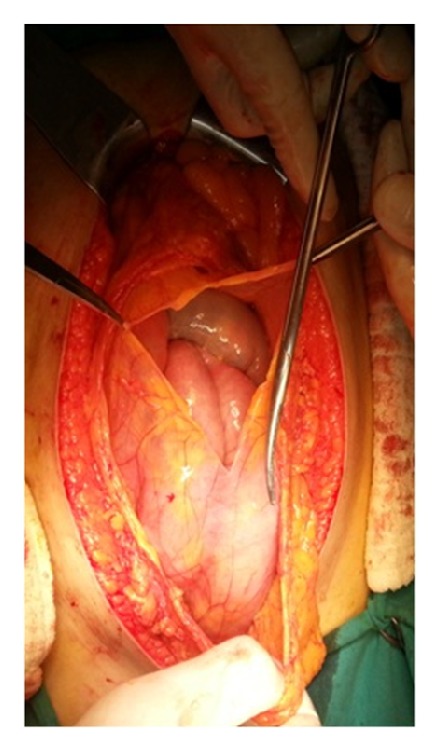
Small intestines located in lesser sac and the surrounding sac.

**Figure 3:**
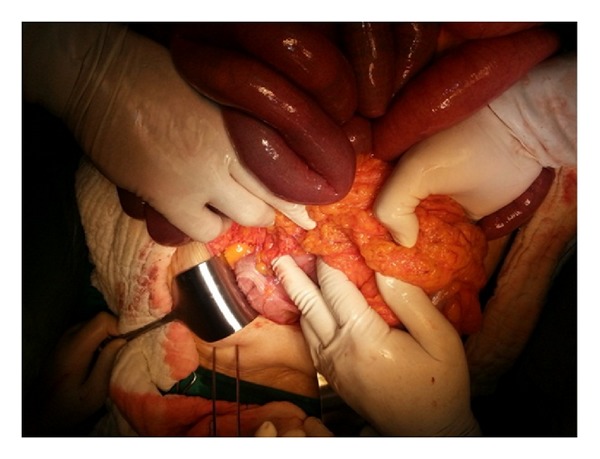
The place where terminal ileum comes out of transverse mesocolon.

A 73-year-old female patient was referred to our department with abdominal pain, swelling, constipation, nausea, and vomiting for 2 days. She had hypertension for 10 years, chronic obstructive pulmonary disease for 6 years, and type 2 diabetes mellitus for 4 years in her medical history. She had no history of abdominal surgery, trauma, jaundice, rectal bleeding, and weight loss. On physical examination, abdominal distension, tinkling, and increased bowel sounds were observed. There was generalised abdominal tenderness, guarding was positive, and rebound was negative. The ampulla was found to be empty on digital rectal examination. In laboratory tests, the leukocyte count was 12,500 K/*μ*L (normal range: 4.6–10.2 K/*μ*L), and the blood glucose level was determined to be 150 mg/dL. Other biochemical tests were within normal limits. Air-fluid levels localised in the upper left quadrant were determined by abdominal X-ray. Ultrasonography could not be performed because of dense intestinal gas. On abdominal tomography, small intestinal segments were observed to be dilated in the left quadrant and distal part of ileum; colon segments were collapsed (Figure 1). The patient underwent surgery with the diagnosis of acute intestinal obstruction. There was no free abdominal fluid at the exploration. The colon was observed in the normal position with lower right localisation of the caecum. The small intestines were palpated under the gastrocolic ligament, which was then opened. The intestines were located in the lesser sac surrounded by a sac, which was opened (Figure 2). The intestines were in a dilated position, and intestinal perfusion was normal. No space-occupying lesion was found. The terminal ileum had entered into the right lower part of the transverse mesocolon (right side of the middle colic artery) and was obstructed there. No cohesiveness or input-output section similar to herniation was found in this transition area. This area was similar to the right localisation ligament of Treitz. After the obstruction, the ileum moved 4 cm further and was joined to the caecum (Figure 3). It is defined as intraperitoneal hernia form of intestinal malrotation [7]. No other small intestinal part in the abdominal cavity except this 4 cm ileum segment was noted. There was no possibility of internal herniation at the point where the terminal ileum passed through the transverse mesocolon. Therefore, it was thought that congenital malrotation caused an obstruction in this case. The obstruction was opened by widening the hole through which the ileum passed in the transverse mesocolon. The intestines were pulled from this aperture to the normal position in the abdominal cavity. The defect in the mesocolon was covered with sutures. The patient started to ingest food orally on the third postoperative day, and she was discharged uneventfully on the fifth day. There was no complaint at the first-month follow-up. The complaint of swelling that had occurred repeatedly for the last 2 years also disappeared.

## 3. Discussion

The intestines are classified into three groups based on the origin of the arterial supplies: foregut, midgut, and hindgut. The duodenum, ileum, jejunum, caecum, and ascending colon constituting two-thirds of the proximal part of the transverse colon are supplied from the superior mesenteric artery (SMA). Intestinal rotation is completed within 4–12 weeks of intrauterine life. The rapid prolongation of the intestine and physiologic herniation into the umbilical cord occurs in the fifth week, a 270° anticlockwise rotation along the SMA axis and the return of herniation back into the abdominal cavity occur in the 10th week, and the location of the caecum in the right lower quadrant are completed in the 12th week [8]. The variations between the normal rotation and failure of the intestines to rotate due to any malfunction in this process are known as malrotations [6]. Although malrotation is a disease in which small intestines located in the right abdominal quadrant and the colon and caecum located in the left quadrant are generally unrotated owing to the bands and adherences [9]. There are several types of malrotation: diversity of anatomic configurations, ranging from a not-quite normal intestinal position to complete nonrotation [7]. The intermediate forms are known as atypical malrotations [6]. The most common variations are nonrotation, reverse fixation, and malrotation [5]. We described that our case was a type intraperitoneal hernia.

The symptoms in newborn infants are intestinal obstruction findings, such as bilious vomiting [10]. Malrotation is generally determined incidentally in adults because it often progresses asymptomatically or with nonspecific mild symptoms. Patients may have crampy abdominal pain, nausea, or bilious vomiting symptoms [5]. Therefore, complete or partial small bowel obstruction and vascular occlusion may develop [11]. The incidence of intestinal malrotation was found to be 0.2% when incidentally found in imaging studies performed for other reasons [3]. The rate of malrotation in autopsies is estimated to be 1 in 6,000. Typical malrotation is a paediatric surgical disease with well-known diagnostic and treatment aspects. Ladd's procedure is the choice of treatment, consisting of volvulus reduction, separation of the abdominal peritoneal bands, and placing of the small intestines in the right quadrant and caecum in the left quadrant of the abdomen [12]. By contrast, atypical malrotation is not a well-defined condition [6]. Asymptomatic cases are often seen in adults explored for other reasons. However, intestinal malrotation is a rare cause of intestinal obstruction in adults. Establishing a diagnosis before the operation is difficult because the symptoms are nonspecific, and malrotation is a rarely seen condition.

Infants and children are diagnosed largely through upper gastrointestinal contrast studies [7]. Adults are diagnosed using various imaging modalities, including upper gastrointestinal contrast studies, barium enema, plain abdominal radiography, computed tomography (CT), and ultrasonography. In adults, plain abdominal radiography may show abnormal localisation of the intestine. Abdominal CT can show clearly bowel settlements and the position of SMA. Surgery for incidentally detected asymptomatic cases is controversial because of the risk of volvulus and obstruction. However, surgery is recommended for patients with intestinal obstruction [5]. Ladd's procedure in nonrotation can be applied with success laparoscopically with a short hospital stay and early recovery benefits [13]. The procedure involves reduction of the volvulus, if present, division of the abnormal peritoneal bands (Ladd's bands), placement of the small bowel to the right of the abdomen and caecum to the left, and appendectomy. We believe that, in other variants of malrotation, considering the volvulus, ischaemia, internal herniation, and obstruction possibilities, as well as case-based surgery (adhesion lyses and placement of the organs more closely to normal anatomy), would be appropriate.

## 4. Conclusion

Intestinal malrotation is not only a newborn disease. Surgeons may encounter malrotations that, in rare cases, can lead to obstruction in adults. In these cases, treating the obstruction and placing the intestines as close as possible to their normal anatomical position may be an appropriate surgical approach.
